# Advanced prokaryotic systematics: the modern face of an ancient science

**DOI:** 10.1016/j.nmni.2022.101036

**Published:** 2022-11-11

**Authors:** Imen Nouioui, Vartul Sangal

**Affiliations:** a Leibniz-Institut DSMZ, Deutsche Sammlung von Mikroorganismen und Zellkulturen GmbH, Inhoffenstraße 7 B, 38124, Braunschweig, Germany; b Faculty of Health and Life Sciences, Northumbria University, Newcastle upon Tyne, NE1 8ST, UK

**Keywords:** Average amino acid identity, average nucleotide identity, digital DNA-DNA hybridisation, Next generation sequencing, percentage of conserved proteins, phylogenomic analyses, prokaryotic systematics, TYGS, Type (Strain) Genome Server, GBDP, Genome BLAST Distance Phylogeny, ANI, Average nucleotide identity, AAI, Average amino acid identity, dDDH, Digital-DNA-DNA hybridisation, POCP, Percentage of conserved proteins, CPR, Candidate Phyla Radiation

## Abstract

Prokaryotic systematics is one of the most progressive disciplines that has embraced technological advances over the last century. The availability and affordability of new sequencing technologies and user-friendly software have revolutionised the discovery of novel prokaryotic taxa, including the identification and nomenclature of uncultivable microorganisms. These advances have enabled scientists to resolve the structure of complex heterogenous taxon and to rectify taxonomic status of misclassified strains due to errors associated with the sensitivity and/or reproducibility of phenotypic approaches. Time- and labour-intensive experimental characterisation of strains could be replaced with determining the presence or absence of genes or operons responsible for phenotypic and chemotaxonomic properties, such as the presence of mycolic acids and menaquinones. However, the quality of genomic data must be acceptable and phylogenomic threshold values for interspecies and supraspecies delineation should be carefully considered in combination of genome-based phylogeny for a reliable and robust classification. These technological developments have empowered prokaryotic systematists to reliably identify novel taxa with an understanding of community ecology and their biosynthetic and biodegradation potentials.

## 1 Introduction

Prokaryotic systematics, a discipline of identifying, classifying and naming novel prokaryotic taxa, provides foundation to all microbiological research of agricultural, ecological, industrial, medical and veterinary importance. Prokaryotes are abundant in nature with an estimated 2.2–4.3 million species, of which only ∼21,000 have been characterised and validly published [[1], [2], [3]]. The discipline that primarily relied on phenotypic characterisation for species identification has evolved over a century since the first edition of Bergey's Manual of Determinative Bacteriology in 1923 to include numerical taxonomy in 1962 and adopt polyphasic taxonomy in 1990s [[3], [4], [5], [6]]. Polyphasic taxonomy including morphological, biochemical and chemotaxonomic characterisation, DNA-DNA hybridisation (DDH) and phylogenetic analysis of variation in the16S rRNA gene sequence has been a gold standard in prokaryotic systematics until whole genome sequencing became affordable in the last decade. Polyphasic systematics is time- and labour-intensive and involve approaches such as DDH that are quite sensitive to minor variations in experimental conditions and probes used in hybridisation, which sometimes results in poor reproducibility between different laboratories [7,8]. The variation in the 16S rRNA gene sequences may not be enough to resolve closely related species [9]. Distinct species can show >99% similarities in their 16S rRNA gene sequences, for example, several species within the family *Geodermatophilaceae* [10] and within the genus *Mycobacterium* [11]. Furthermore, horizontal transfer of 16S rRNA gene has been reported in multiple bacterial species [[12], [13], [14]], suggesting that variation in 16S rRNA gene should be carefully considered for species delineation.

To overcome these limitations, prokaryotic systematists have embraced and integrated whole genome sequence-based analyses in their practices [3,[15], [16], [17], [18], [19], [20]]. Not only it resolved the taxonomic status of complex taxon [[17], [18], [19],^21^,^22^], but it is also now possible to define new species of unculturable prokaryotes based on single-amplified genomes and metagenome-assembled genomes [[23], [24], [25]].

The modernisation of the practices of prokaryotic systematics was not possible without significant contribution and tireless efforts of the scientific community continuously developing bioinformatic tools to help systematists with limited computational experience. The availability of interactive user-friendly bioinformatics tools and resources played an important role in facilitating the smooth integration of whole genome sequences into the discipline. For example, EzBioCloud and Type (Strain) Genome Server (TYGS) are extensively used resources by prokaryotic systematists. EzBioCloud has an integrated sequence database of high-quality 16S rRNA gene and genome sequences of archaea and bacteria [26]. The server hosts various bioinformatic tools for identification of novel species from 16S rRNA or genome sequences. Similarly, TYGS is a simple and fully automated genome analysis pipeline that can extract 16S rDNA gene sequences from the genome sequences and determine the closest type strains for further analyses [27,28]. TYGS also performs state-of-the-art phylogenomic analysis on a user provided set of genomes and constructs a highly robust genome-based phylogenetic tree using the Genome BLAST Distance Phylogeny (GBDP) method [27,28]. However, there are still some challenges that remains to be addressed. In this review, we discuss the progress made by the incorporation of genomics into prokaryotic systematics along with important computational tools, and how the definition of genus and species evolved over the last decade.

## 2 Important phylogenomic approaches for prokaryotic systematics

Several taxogenomic approaches are available to assist systematists in identification of novel taxon by comparing genome sequences. One of the most used approaches is the calculation of average nucleotide identity (ANI) between pairs of genomes where genome sequences are spliced into 1020 bp fragments followed by BLAST-search [29] against other genomes [30,31]. ANI values are calculated from pairwise matches with ≥70% alignable length and at least 30% sequence identity. The users could easily compare up to 50 genomes by using online Genome-based distance matrix calculator (http://enve-omics.ce.gatech.edu/g-matrix/). OrthoANI is another great tool that calculates ANI values from a set of orthologues genes within the dataset to address the bias introduced by the variation in the size of sequence alignments by pairwise reciprocal similarity criteria [32]. However, both these approaches could be slower for large datasets. More recently, a fast computational approach has been developed that calculates ANI values from orthologous gene pairs between two genomes using an alignment-free approximate sequence mapping method [33]. The reliability of all these approaches seems to be comparable with a cut-off value of 95% for defining species [30,32,33].

Pairwise average amino acid identity (AAI) values are often treated to be more reliable, particularly for circumscribing higher taxa [31,34,35]. AAI values are calculated from the protein sequences showing >30% identity and an alignable region of >70% after a two-way BLAST search [36,37]. Again, the online Genome-based distance matrix calculator (http://enve-omics.ce.gatech.edu/g-matrix/) with a user-friendly interface could be used to calculate AAI values for up to 50 genomes. Another efficient and easy to use tool for AAI calculation is EzAAI pipeline, which uses hierarchical clustering tool MMseqs2 [38] for a sensitive protein sequence search and faster calculation of AAI values [39]. The AAI values were comparable to those obtained from BLAST based approach. Similar to ANI, 95% threshold is used for defining species.

An important approach for species delineation is the calculation of digital-DDH (dDDH) values using an efficient user-friendly web interface, genome-to-genome distance calculator [40,41]. The calculator will estimate overall similarity between pair of genomes and provide a value that mimics the experimental DDH values, helping systematists to avoid tedious and often irreproducible laboratory-based DNA-DNA re-association experimentation. Similar to the laboratory-based experimental DDH values, two strains with ≥70% dDDH value are assigned to the same species. The values are calculated by three different formulas but formula #2 is recommended that is not affected by the size of genomes and provides robust values, particularly if incomplete draft genomes are analysed [42].

A pangenomic approach that is based on the calculation of the percentage of the core/pan-genome ratio between different strains is emerging as an important tool for reclassification of bacterial species [43,44]. Distinct species were estimated to show a discontinuity or an abrupt break of >10% in the core/pan-genome ratio [43,44]. The core/pan-genome ratio was 94% when six *Klebsiella pneumoniae* genomes were compared; however, this ratio was decreased to 79% and 72% when *Klebsiella pneumoniae* subsp. *rhinoscleromatis* and *K. pneumoniae* subsp. ozaenae were successively compared [43]. A reduction of 13-27% was observed when *Klebsiella mobilis*, *Klebsiella variicola* and *Klebsiella oxytoca* genomes were compared with the six *Klebsiella pneumoniae* genomes, leading to the conclusion that *K. pneumoniae* subsp. *rhinoscleromatis* and *K. pneumoniae* subsp. *ozaenae* should be treated as distinct species [[43], [44], [45]].

A potential ANI threshold of ∼75% has been suggested for separating different genera [17]. However, the suitability of ANI values in delineating genera was challenged in favour of the percentage of conserved proteins (POCP) between a pair of genomes [46]. The conserved proteins are identified based on >50% aligned length with >40% sequence identity and an e-value <1 × 10^−5^. The hypothesis is that at least 50% of the genome wide proteome will be shared by two individual species belonging to the same genus [46].

Phylogenetic analyses are important to infer evolutionary relatedness among different strains that help systematists in assigning species to correct taxon. PhyloPhlAn 3.0 is a highly efficient approach to rapidly construct phylogenies from large genomic datasets [47]. The program automatically selects the most informative clade-specific loci from genome assemblies or genome-wide protein sequences to create multiple-sequence alignment and construct a maximum-likelihood tree [47]. PhyloPhlAn trees are highly robust and can perform taxonomic assignments based on the NCBI taxonomy. However, several other approaches including multilocus sequence analyses, core genome phylogeny and genome distance-based phylogeny [40,48] have also been used to study taxonomic relatedness between different strains. 16S rRNA sequences remain central to the process that helps identify relevant genomes/organisms for comparative analyses. GenBank is the fastest growing database of prokaryotic genomes (363,287 prokaryotic genome sequences; accessed on 01/10/2021) that has served as a great resource for facilitating phylogenomic comparisons [49]. The scientific community can freely access the genome sequences and can use them in comparative analyses. As mentioned before, automated genomic pipelines such as EzBioCloud server [26] and TYGS [28] have integrated phylogenetic and taxogenomic approaches that are interactive and very easy to use for determining taxonomic status of strains and to classify them without the need of installing any software. The availability of easy-to-use computational tools contributed to the integration of genome sequencing into prokaryotic systematics.

## 3 What has been resolved by integration of genomics into prokaryotic systematics?

The taxonomic structure of large complex taxon, such as the phylum *Actinomycetota* (former known as *Actinobacteria*), remained unresolved until the integration of genomics [19]. The phylogenomic analyses of 1,142 strains led to a large scale restructuring of the phylum including the addition of two new orders, ten families, 17 new genera along with the reclassification of more than 100 species [19]. *Rhodococcus*, a heterogeneous actinobacterial taxon that include species of ecological, industrial and medical importance, encompasses multiple species-groups which equate to the rank of distinct genera [17,22]. A phylogenomic review of the phylum *Bacteroidota* (formerly known as *Bacteroidetes*) resulted in elevation of the families *Balneolaceae* and *Saprospiraceae* to a new phylum *Balneolaeota,* and addition of a new class *Saprospiria* [50]. Similarly, phylogenomic analyses of the genus *Flexibacter* resulted in defining new monophyletic genera with emended species descriptions [50].

Phylogenomic analyses also resolved the taxonomic structure of clinically and ecologically important taxa. The genus *Mycobacterium sensu lato* was divided into five monophyletic clades designated as *“Fortuitum-Vaccae”, “Terrae*”*, “Triviale*”*, “Abscessus-Chelonae*” and "*Tuberculosis-Simiae",* which correspond to four novel genera *Mycolicibacterium, Mycolicibacter, Mycolicibacillus, Mycobacteroides,* respectively [51]*.* The "*Tuberculosis-Simiae"* clade encompasses pathogenic strains including *Mycobacterium tuberculosis*, which is responsible for tuberculosis in humans. Similarly, *Burkholderia cepacian* complex containing strains that are responsible for fatal lung infection in humans, was resolved into 36 groups with a reassignment of 22 species to the correct rank and identification of 14 novel species [52].

The genus *Frankia* is known for ecological and agricultural significance but the taxonomic relatedness between *Frankia* species remained vague [53,54]. Using phylogenomic analyses, twelve *Frankia* species were shown to form four distinct groups with capacities to produce plant growth promoting hormones, bioremediation potential, and potential to produce novel bioactive compounds [20]. Phylogenomic analyses also highlighted close phylogenetic relationship of the genera *Streptomyces, Kitasatospora* and *Streptoacidiphilus*, which are well known for their ecological and genetic diversity [55]. These genera shared a number of genomic features and capacities to produce similar secondary metabolites, potentially due to high recombination frequencies [55]. In addition, genomic analysis provided new insights into biotechnological potential of *Kitasatospora* for novel antibiotic discovery [55]. Therefore, integration of phylogenomics into prokaryotic systematics made the classification of prokaryotic taxa more robust, helped understand evolutionary processes behind the genomic diversity between related taxa and revealed biosynthetic capacities of different strains.

## 4 Time to move on to the modern prokaryotic systematics

Traditional phenotyping methods using *in vitro* biochemical assays or API 20/CHL strips provide an overview of the metabolic features of microorganisms but are sometimes sensitive to minor variations in experimental conditions, resulting in poor reproducibility and/or ambiguous data. But do we really need to continue with the traditional phenotyping methods?

Some studies reported a lack of consistency between chemotaxonomic and phylogenetic data; for example, the genus *Turicella* which only differed from *Corynebacterium* strains by lacking mycolic acids and quinone profiles [19,56]. Genomic characterisation revealed that chemotaxonomic variations were contributed by deletion of genes responsible for synthesis of mycolic acids and menaquinone in the former [57]. *Turicella otitidis*, the sole *Turicella* species, was reclassified as *Corynebacterium otitidis* based on phylogenetic analyses [57]. Therefore, identification of genetic basis of phenotypic characteristics could replace phenotypic and chemotaxonomic characterisation of microorganisms. It is now possible to model the pathways for a particular phenotype by mapping them onto the genome sequences which is emerging as a prominent discipline called microbial phenomics [58].

High throughput phenotyping technologies such as Biolog Phenotype MicroArrays, have been extensively used for characterisation of metabolic pathways in combination with the genomic data, which has also been useful for taxonomic studies [53,[59], [60], [61]]. This system can determine approximately 2,000 phenotypes for a microbial cell including their capacities to use different carbon, nitrogen, phosphorus and sulphur substrates, abilities to tolerate a range of pH and salinity, and sensitivity to several antibiotics and inhibitory compounds. Several R packages are available for processing of high-throughput phenotypic data [62,63]. Therefore, it is time to modernise the practices of polyphasic taxonomy by minimising the battery of biochemical and chemotaxonomic assays and adopting a more balanced taxonogenomic approach for species description. Taxonogenomics combines phenotypic and genotypic characteristics for identification of microorganisms [64]. The most relevant basic features, such as morphological and growth properties, along with the phylogenomic characteristics should be sufficient for a species description [[64], [65], [66], [67]]. Indeed, several new species have been described using the taxonogenomic approach [[68], [69], [70]].

## 5 Has genomics empowered prokaryotic systematists?

Despite the importance of non-cultivable microbes in maintaining different ecosystems, our knowledge of the microbial world is mostly limited to cultivable taxa. Only ∼2% bacteria from nearly half of the identifiable bacterial domains are culturable [71]. Environmental metagenomics and single cell sequencing made it possible to assess the microbial diversity including the unculturable bacteria, so-called ‘microbial dark matter’, in their ecological niches, which is guiding further studies to investigate their biosynthetic or catalytic potential [[72], [73], [74]]. New taxa identified by culture-independent methods have expanded the tree of life to include “Candidate Phyla Radiation (CPR)”, a large group of mostly non-cultivable bacterial lineages that represent more than 15% of the domain Bacteria, including representatives of over 70 phyla and two superphyla (*Candidatus* Parcubacteria and *Candidatus* Microgenomates) [75]. Significantly higher numbers of new phyla and novel species are regularly being identified from complex environmental samples using metagenomic and single cell sequencing approaches in comparison to traditional culture-based approach [[76], [77], [78]]. In line with the practices of prokaryotic systematics, a roadmap for nomenclature of high numbers of nonculturable Archaea and Bacteria has been developed and is extensively discussed by the community [24]. Assessing the cell number and morphology of non-culturable bacteria from a complex sample is also now possible by using microfluidic single-cell dispenser systems [79]. Therefore, these great technological developments have empowered systematists not only to identify novel prokaryotic taxa but also to study community ecology and their biosynthetic and catalytic (biodegradation) capacities.

## 6 The key challenges

The technological advancements and availability of user-friendly phylogenomic tools have clearly benefited prokaryotic systematists. However, there are still some challenges, which need addressing. The most important issue is the quality of the sequencing reads and assembled genomes. More than 2 million entries in the GenBank were found to be contaminated in a recent study, including 4-8% of data for bacterial species [80]. It is important to check the quality of sequencing reads and genome assemblies before using them in phylogenomic analyses. Too many short contigs and those with poor coverage should be excluded. Genome assemblies should be checked for completeness, contamination and percentage of ambiguous bases using the programs such as CheckM [81] before downstream analyses.

While phylogenomic tools provide usable matrices, the threshold values suggested for interspecies and supraspecies delineation should be carefully considered in combination of genome-based phylogeny [[82], [83], [84]]. For instance, if dDDH values are marginally below the threshold and ANI and/or AAI values are >95% for a pair of genomes, phylogenetic relationship could be considered along with other ecological characteristics, such as association with a particular clinical condition that has been characterised with the presence of the same set of virulence genes. In a recent study, *Corynebacterium* strains from yellow-eyed penguins belonging to two distinct lineages were proposed to be assigned to two subspecies despite dDDH values being below the 70% threshold due to ANI and AAI values that were higher the 95% cut-off, very close phylogenetic relationship and their association with diphtheritic-stomatitis [84].

The publicly available resources allow users to analyse smaller number of genomes that are often limited to 50-75 genomes. It causes problems for scientists resolving the taxonomic status of large and complex taxon where these programs need to be installed on Unix/Linux based high-performance computers and data needs to be analysed using the command lines. It requires significant investment in computational resources and advance knowledge to configure and run phylogenomic analyses that may not be feasible for all prokaryotic systematists.

## 7 Conclusions - The “genomic” definition of a genus and species

According to the polyphasic approach, groups of phylogenetically closely related strains with similar phenotypic characteristics, ≥70% pairwise DNA-DNA hybridization (DDH) values and >98.7% identity between their 16S rRNA gene sequences are often assigned to the same species [[85], [86], [87]]. The definition of prokaryotic genera is a bit vague that often relies on monophyly of strains in phylogenetic tree and average rRNA gene sequence divergence <6%.

Phylogenomically, a prokaryotic genus is defined as a monophyletic group of species sharing at least 50% of genome wide proteome (pairwise POCP values ≥ 50%). A group of phylogenetically closely related strains with ≥95% ANI and/or AAI values and dDDH value of ≥70% belongs to the same species. Therefore, genome based phylogenomic metrices could be used for interspecies and supraspecies delineation.

In summary, prokaryotic systematics is clearly a progressive discipline that has evolved over a century by embracing advanced technologies. While polyphasic approach provides important information on the cellular composition and lifestyle of an organism, these techniques sometimes show poor reproducibility between different laboratories. Since whole genome sequencing has integrated well into prokaryotic systematics, more emphasis could be placed on describing the genetics, *i.e*., presence or absence of genes involved in phenotypic properties, where possible. It is probably the time to accept the phylogenomic description of novel taxa (species and genera) for validation where quality of genome assemblies is excellent and robust phylogenomic analyses present compelling evidence without extensive polyphasic characterisation (phenotypic and chemotaxonomic properties).

## CRediT authorship contribution statement

**Imen Nouioui:** Data curation, Writing – original draft. **Vartul Sangal:** Conceptualization, Data curation, Writing – original draft.

## References

[1] Louca S., Mazel F., Doebeli M., Parfrey L.W. (2019). A census-based estimate of Earth's bacterial and archaeal diversity. PLoS Biol.

[2] Parks D.H., Chuvochina M., Chaumeil P.A., Rinke C., Mussig A.J., Hugenholtz P. (2020). A complete domain-to-species taxonomy for Bacteria and Archaea. Nat Biotechnol.

[3] Hugenholtz P., Chuvochina M., Oren A., Parks D.H., Soo R.M. (2021). Prokaryotic taxonomy and nomenclature in the age of big sequence data. ISME J.

[4] Bergey D.H., Harrison F.C., Breed R.S., Hammer B.W., Huntoon F.M. (1923).

[5] Sneath P.H., Sokal R.R. (1962). Numerical taxonomy. Nature.

[6] Vandamme P., Pot B., Gillis M., de Vos P., Kersters K., Swings J. (1996). Polyphasic taxonomy, a consensus approach to bacterial systematics. Microbiol Rev.

[7] Achtman M., Wagner M. (2008). Microbial diversity and the genetic nature of microbial species. Nat Rev Microbiol.

[8] Moore E.R., Mihaylova S.A., Vandamme P., Krichevsky M.I., Dijkshoorn L. (2010). Microbial systematics and taxonomy: relevance for a microbial commons. Res Microbiol.

[9] Park G., Jin W.Y., Jang S.J., Kook J.K., Choi J.A., Park G.C. (2015). Evaluation of four methods of assigning species and genus to medically important bacteria using 16S rRNA gene sequence analysis. Microbiol Immunol.

[10] Montero-Calasanz M.D.C., Meier-Kolthoff J.P., Zhang D.F., Yaramis A., Rohde M., Woyke T. (2017). Genome-scale data call for a taxonomic rearrangement of *Geodermatophilaceae*. Front Microbiol.

[11] Beye M., Fahsi N., Raoult D., Fournier P.E. (2018). Careful use of 16S rRNA gene sequence similarity values for the identification of *Mycobacterium* species. New Microbe New Infect.

[12] Kitahara K., Miyazaki K. (2013). Revisiting bacterial phylogeny: natural and experimental evidence for horizontal gene transfer of 16S rRNA. Mob Genet Elem.

[13] Sato M., Miyazaki K. (2017). Phylogenetic network analysis revealed the occurrence of horizontal gene transfer of 16S rRNA in the genus *Enterobacter*. Front Microbiol.

[14] Schouls L.M., Schot C.S., Jacobs J.A. (2003). Horizontal transfer of segments of the 16S rRNA genes between species of the *Streptococcus anginosus* group. J Bacteriol.

[15] Chun J., Rainey F.A. (2014). Integrating genomics into the taxonomy and systematics of the *Bacteria* and *Archaea*. Int J Syst Evol Microbiol.

[16] Sangal V., Nieminen L., Tucker N.P., Hoskisson P.A., Goodfellow M., Sutcliffe I.C., Chun J. (2014). Methods in microbiology: bacterial taxonomy.

[17] Sangal V., Goodfellow M., Jones A.L., Schwalbe E.C., Blom J., Hoskisson P.A. (2016). Next-generation systematics: an innovative approach to resolve the structure of complex prokaryotic taxa. Scientific Rep.

[18] Sangal V., Goodfellow M., Blom J., Tan G.Y.A., Klenk H.P., Sutcliffe I.C. (2018). Revisiting the taxonomic status of the biomedically and industrially important genus *Amycolatopsis*, using a phylogenomic approach. Front Microbiol.

[19] Nouioui I., Carro L., García-López M., Meier-Kolthoff J.P., Woyke T., Kyrpides N.C. (2018). Genome-based taxonomic classification of the phylum *Actinobacteria*. Front Microbiol.

[20] Nouioui I., Cortes-albayar C., Carro L., Castro J.F., Gtari M., Ghodhbane-Gtari F. (2019). Genomic insights into plant-growth-promoting potentialities of the genus *Frankia*. Front Microbiol.

[21] Carro L., Nouioui I., Sangal V., Meier-Kolthoff J.P., Trujillo M.E., Montero-Calasanz M.D.C. (2018). Genome-based classification of micromonosporae with a focus on their biotechnological and ecological potential. Scientific Rep.

[22] Sangal V., Goodfellow M., Jones A.L., Seviour R.J., Sutcliffe I.C., Alvarez H. (2019). Biology of *Rhodococcus*.

[23] Hedlund B.P., Dodsworth J.A., Staley J.T. (2015). The changing landscape of microbial biodiversity exploration and its implications for systematics. Syst Appl Microbiol.

[24] Murray A.E., Freudenstein J., Gribaldo S., Hatzenpichler R., Hugenholtz P., Kampfer P. (2020). Roadmap for naming uncultivated Archaea and Bacteria. Nat Microbiol.

[25] Picone N., Blom P., Hogendoorn C., Frank J., van Alen T., Pol A. (2021). Metagenome assembled genome of a novel verrucomicrobial methanotroph from Pantelleria Island. Front Microbiol.

[26] Yoon S.H., Ha S.M., Kwon S., Lim J., Kim Y., Seo H. (2017). Introducing EzBioCloud: a taxonomically united database of 16S rRNA gene sequences and whole-genome assemblies. Int J Syst Evol Microbiol.

[27] Meier-Kolthoff J.P., Carbasse J.S., Peinado-Olarte R.L., Goker M. (2022). TYGS and LPSN: a database tandem for fast and reliable genome-based classification and nomenclature of prokaryotes. Nucleic Acids Res.

[28] Meier-Kolthoff J.P., Goker M. (2019). TYGS is an automated high-throughput platform for state-of-the-art genome-based taxonomy. Nat Commun.

[29] Camacho C., Coulouris G., Avagyan V., Ma N., Papadopoulos J., Bealer K. (2009). BLAST+: architecture and applications. BMC Bioinform.

[30] Goris J., Konstantinidis K.T., Klappenbach J.A., Coenye T., Vandamme P., Tiedje J.M. (2007). DNA-DNA hybridization values and their relationship to whole-genome sequence similarities. Int J Syst Evol Microbiol.

[31] Rodriguez-Rivera L.D., Konstantinidis K.T. (2014). Bypassing cultivation to identify bacterial species. ASM Microbe Magazine.

[32] Lee I., Ouk Kim Y., Park S.C., Chun J. (2016). OrthoANI: an improved algorithm and software for calculating average nucleotide identity. Int J Syst Evol Microbiol.

[33] Jain C., Rodriguez R.L., Phillippy A.M., Konstantinidis K.T., Aluru S. (2018). High throughput ANI analysis of 90K prokaryotic genomes reveals clear species boundaries. Nat Commun.

[34] Richter M., Rosselló-Móra R. (2009). Shifting the genomic gold standard for the prokaryotic species definition. Proc Natl Acad Sci U S A.

[35] Rosselló-Móra R., Amann R. (2015). Past and future species definitions for Bacteria and Archaea. Syst Appl Microbiol.

[36] Konstantinidis K.T., Tiedje J.M. (2005). Genomic insights that advance the species definition for prokaryotes. Proc Natl Acad Sci U S A.

[37] Konstantinidis K.T., Tiedje J.M. (2005). Towards a genome-based taxonomy for prokaryotes. J Bacteriol.

[38] Steinegger M., Soding J. (2017). MMseqs2 enables sensitive protein sequence searching for the analysis of massive data sets. Nat Biotechnol.

[39] Kim D., Park S., Chun J. (2021). Introducing EzAAI: a pipeline for high throughput calculations of prokaryotic average amino acid identity. J Microbiol.

[40] Meier-Kolthoff J.P., Auch A.F., Klenk H.P., Goker M. (2013). Genome sequence-based species delimitation with confidence intervals and improved distance functions. BMC Bioinform.

[41] Meier-Kolthoff J.P., Klenk H.P., Goker M. (2014). Taxonomic use of DNA G+C content and DNA-DNA hybridization in the genomic age. Int J Syst Evol Microbiol.

[42] Auch A.F., Klenk H.P., Göker M. (2010). Standard operating procedure for calculating genome-to-genome distances based on high-scoring segment pairs. Stand Genomic Sci.

[43] Caputo A., Merhej V., Georgiades K., Fournier P.E., Croce O., Robert C. (2015). Pan-genomic analysis to redefine species and subspecies based on quantum discontinuous variation: the *Klebsiella* paradigm. Biol Direct.

[44] Caputo A., Fournier P.E., Raoult D. (2019). Genome and pan-genome analysis to classify emerging bacteria. Biol Direct.

[45] Janda J.M., Abbott S.L. (2021). The changing face of the family *Enterobacteriaceae* (Order: "*Enterobacterales*"): new members, taxonomic issues, geographic expansion, and new diseases and disease syndromes. Clin Microbiol Rev.

[46] Qin Q.L., Xie B.B., Zhang X.Y., Chen X.L., Zhou B.C., Zhou J. (2014). A proposed genus boundary for the prokaryotes based on genomic insights. J Bacteriol.

[47] Asnicar F., Thomas A.M., Beghini F., Mengoni C., Manara S., Manghi P. (2020). Precise phylogenetic analysis of microbial isolates and genomes from metagenomes using PhyloPhlAn 3.0. Nat Commun.

[48] Auch A.F., Henz S.R., Holland B.R., Goker M. (2006). Genome BLAST distance phylogenies inferred from whole plastid and whole mitochondrion genome sequences. BMC Bioinform.

[49] Sayers E.W., Beck J., Bolton E.E., Bourexis D., Brister J.R., Canese K. (2021). Database resources of the national center for biotechnology information. Nucleic Acids Res.

[50] Hahnke R.L., Meier-Kolthoff J.P., Garcia-Lopez M., Mukherjee S., Huntemann M., Ivanova N.N. (2016). Genome-based taxonomic classification of *Bacteroidetes*. Front Microbiol.

[51] Gupta R.S., Lo B., Son J. (2018). Phylogenomics and comparative genomic studies robustly support division of the genus *Mycobacterium* into an emended genus *Mycobacterium* and four novel genera. Front Microbiol.

[52] Jin Y., Zhou J., Zhou J., Hu M., Zhang Q., Kong N. (2020). Genome-based classification of *Burkholderia cepacia* complex provides new insight into its taxonomic status. Biol Direct.

[53] Gtari M., Nouioui I., Sarkar I., Ghodhbane-Gtari F., Tisa L.S., Sen A. (2019). An update on the taxonomy of the genus *Frankia* Brunchorst, 1886, 174(AL). Antonie Van Leeuwenhoek.

[54] Gtari M., Tisa L.S., Normand P., Aroca R. (2013). Symbiotic endophytes.

[55] Li Y., Wang M., Sun Z.-Z., Xie B.-B. (2021). Comparative genomic insights into the taxonomic classification, diversity, and secondary metabolic potentials of *Kitasatospora*, a genus closely related to *Streptomyces*. Front Microbiol.

[56] Funke G., Stubbs S., Altwegg M., Carlotti A., Collins M.D. (1994). *Turicella otitidis* gen. nov., sp. nov., a coryneform bacterium isolated from patients with otitis media. Int J Syst Bacteriol.

[57] Baek I., Kim M., Lee I., Na S.I., Goodfellow M., Chun J. (2018). Phylogeny trumps chemotaxonomy: a case study involving *Turicella otitidis*. Front Microbiol.

[58] Thessen A.E., Walls R.L., Vogt L., Singer J., Warren R., Buttigieg P.L. (2020). Transforming the study of organisms: phenomic data models and knowledge bases. PLoS Comput Biol.

[59] Bochner B.R., Gadzinski P., Panomitros E. (2001). Phenotype microarrays for high-throughput phenotypic testing and assay of gene function. Genome Res.

[60] Bochner B.R. (2009). Global phenotypic characterization of bacteria. FEMS Microbiol Rev.

[61] Nouioui I., Ghodhbane-Gtari F., Montero-Calasanz M.D.C., Goker M., Meier-Kolthoff J.P., Schumann P. (2016). Proposal of a type strain for *Frankia alni* (Woronin 1866) Von Tubeuf 1895, emended description of *Frankia alni*, and recognition of *Frankia casuarinae* sp. nov. and *Frankia elaeagni* sp. nov. Int J Syst Evol Microbiol.

[62] Vaas L.A., Sikorski J., Michael V., Goker M., Klenk H.P. (2012). Visualization and curve-parameter estimation strategies for efficient exploration of phenotype microarray kinetics. PLoS One.

[63] Vehkala M., Shubin M., Connor T.R., Thomson N.R., Corander J. (2015). Novel R pipeline for analyzing biolog phenotypic MicroArray data. PLoS One.

[64] Fournier P.E., Drancourt M. (2015). New Microbes New Infections promotes modern prokaryotic taxonomy: a new section "TaxonoGenomics: new genomes of microorganisms in humans. New Microbe New Infect.

[65] Vandamme P., Peeters C. (2014). Time to revisit polyphasic taxonomy. Antonie Van Leeuwenhoek.

[66] Sutcliffe I.C., Trujillo M.E., Goodfellow M. (2012). A call to arms for systematists: revitalising the purpose and practices underpinning the description of novel microbial taxa. Antonie Van Leeuwenhoek.

[67] Sutcliffe I.C., Trujillo M.E., Whitman W.B., Goodfellow M. (2013). A call to action for the international committee on systematics of prokaryotes. Trends Microbiol.

[68] Ben Khedher M., Lo C.I., Diop K., Morand A., Armstrong N., Raoult D. (2021). Taxonogenomics description of *Arcanobacterium urinimassiliense* sp. nov., a new bacterial species isolated from urine sample. New Microbe New Infect.

[69] Sarr M., Diouf F.S., Lo C.I., Tidjani Alou M., Alibar S., Million M. (2021). Taxonogenomics description of *Bacillus marasmi* sp. nov., a new species isolated from the stool sample. New Microbe New Infect.

[70] Bilen M., Mbogning Fonkou M.D., Khelaifia S., Tomei E., Cadoret F., Daoud Z. (2019). Taxonogenomics description of *Parabacteroides timonensis* sp. nov. isolated from a human stool sample. Microbiologyopen.

[71] Rappe M.S., Giovannoni S.J. (2003). The uncultured microbial majority. Annu Rev Microbiol.

[72] Chen S.C., Budhraja R., Adrian L., Calabrese F., Stryhanyuk H., Musat N. (2021). Novel clades of soil biphenyl degraders revealed by integrating isotope probing, multi-omics, and single-cell analyses. ISME J.

[73] Costa O.Y.A., de Hollander M., Pijl A., Liu B., Kuramae E.E. (2020). Cultivation-independent and cultivation-dependent metagenomes reveal genetic and enzymatic potential of microbial community involved in the degradation of a complex microbial polymer. Microbiome.

[74] Charlop-Powers Z., Owen J.G., Reddy B.V., Ternei M.A., Guimaraes D.O., de Frias U.A. (2015). Global biogeographic sampling of bacterial secondary metabolism. eLife.

[75] Rinke C., Schwientek P., Sczyrba A., Ivanova N.N., Anderson I.J., Cheng J.F. (2013). Insights into the phylogeny and coding potential of microbial dark matter. Nature.

[76] Anantharaman K., Brown C.T., Hug L.A., Sharon I., Castelle C.J., Probst A.J. (2016). Thousands of microbial genomes shed light on interconnected biogeochemical processes in an aquifer system. Nat Commun.

[77] Hernsdorf A.W., Amano Y., Miyakawa K., Ise K., Suzuki Y., Anantharaman K. (2017). Potential for microbial H2 and metal transformations associated with novel bacteria and archaea in deep terrestrial subsurface sediments. ISME J.

[78] Idris H., Goodfellow M., Sanderson R., Asenjo J.A., Bull A.T. (2017). Actinobacterial rare biospheres and dark matter revealed in habitats of the Chilean atacama desert. Scientific Rep.

[79] Wiegand S., Dam H.T., Riba J., Vollmers J., Kaster A.K. (2021). Printing microbial dark matter: using single cell dispensing and genomics to investigate the Patescibacteria/Candidate Phyla Radiation. Front Microbiol.

[80] Steinegger M., Salzberg S.L. (2020). Terminating contamination: large-scale search identifies more than 2,000,000 contaminated entries in GenBank. Genome Biol.

[81] Parks D.H., Imelfort M., Skennerton C.T., Hugenholtz P., Tyson G.W. (2015). CheckM: assessing the quality of microbial genomes recovered from isolates, single cells, and metagenomes. Genome Res.

[82] Li X., Huang Y., Whitman W.B. (2015). The relationship of the whole genome sequence identity to DNA hybridization varies between genera of prokaryotes. Antonie Van Leeuwenhoek.

[83] Riesco R., Carro L., Roman-Ponce B., Prieto C., Blom J., Klenk H.P. (2018). Defining the species *Micromonospora saelicesensis* and *Micromonospora noduli* under the framework of genomics. Front Microbiol.

[84] Saunderson S.C., Nouioui I., Midwinter A.C., Wilkinson D.A., Young M.J., McInnes K.M. (2021). Phylogenomic characterization of a novel *Corynebacterium* species associated with fatal diphtheritic stomatitis in endangered yellow-eyed penguins. mSystems.

[85] Kim M., Oh H.S., Park S.C., Chun J. (2014). Towards a taxonomic coherence between average nucleotide identity and 16S rRNA gene sequence similarity for species demarcation of prokaryotes. Int J Syst Evol Microbiol.

[86] Oren A., Garrity G.M. (2014). Then and now: a systematic review of the systematics of prokaryotes in the last 80 years. Antonie Van Leeuwenhoek.

[87] Sneath P.H.A., Boone D.R., Castenholz R.W., Garrity G.M. (2001). Bergey’s manual of systematic Bacteriology.

